# Preventing fire and burn among children in Malaysia: a research driven education initiative

**Published:** 2019-08

**Authors:** Kulanthayan KC Mani, Anusooya Demado, Rosliza Abdul Manaf, Kaviyarasu Yellappan

**Affiliations:** ^*a*^Safe Kids Malaysia Universiti Putra Malaysia, Malaysia.; ^*b*^Department of Community Health, Universiti Putra Malaysia, Malaysia.

## Abstract

**Background:**

Burn injuries are a serious public health problem globally with alarming high mortality and morbidity especially among children and Malaysia in not exceptional to it where we lose one child every fourteen days from a burn injury which is preventable. The aim of this exercise was to understand the magnitude of the Fire and Burn Injury problem and followed by developing and implementing an intervention program as a solution.

**Methods:**

The study was a cross-sectional survey among 640 parents of children attending primary schools (children age 7-12) in Sentul, Kuala Lumpur. A total of 9 primary schools out of 558 were chosen for this study. Questionnaires for the parents were given and collected through the identified children.

**Results:**

Prevalence of fire in the past two years were 6.4% and burn injury among children were alarmingly standing at 54%. The top five frequent occurring cause of burn injuries reported by the parents for their children were: touching hot utensils (24.4%), hot iron (21.7%), hot water heater (17.2%), motorcycle hot exhaust pipe (16.7%) and playing with fire crackers (14.4%).

**Conclusions:**

In conclusion, an educational intervention program on preventing fire and burn was developed based on the top five causes of burn and also focusing on what need to be done in an event of fire as well as injury. The four module education program was intervened to the 6000 primary school children for a duration of 180 minutes combining both classroom teaching session and field demonstration on how to escape and fight fire with the assistance from Fire and Rescue Department as our program partner. The initiative was evaluated and showed an increase in the knowledge score on children understanding on factors causing fire, burn and scald injury. In addition, knowledge score also increased on children understanding on how to response in an event of fire. With the success of the pilot program, it is highly hoped all relevant stakeholders and funders could join hand together in scaling up the intervention to reach out to more school children.

**Keywords:**

Children, Fire, Burn, Research and Education Initiative

